# Natural Selection and Scale Invariance

**DOI:** 10.3390/life13040917

**Published:** 2023-03-31

**Authors:** Adrian F. Tuck

**Affiliations:** Retired Scientist, 3401 Arapahoe Avenue Unit 317, Boulder, CO 80303, USA; adrianftuck@gmail.com; Tel.: +1-303-506-9205

**Keywords:** molecular dynamics, statistical multifractals, non-equilibrium thermodynamics, Gibbs free energy, symmetry breaking

## Abstract

This review points out that three of the essential features of natural selection—competition for a finite resource, variation, and transmission of memory—occur in an extremely simple, thermalized molecular population, one of colliding “billiard balls” subject to an anisotropy, a directional flux of energetic molecules. The emergence of scaling behavior, scale invariance, in such systems is considered in the context of the emergence of complexity driven by Gibbs free energy, the origins of life, and known chemistries in planetary and astrophysical conditions. It is suggested that the thermodynamic formalism of statistical multifractality offers a parallel between the microscopic and macroscopic views of non-equilibrium systems and their evolution, different from, empirically determinable, and therefore complementing traditional definitions of entropy and its production in living systems. Further, the approach supports the existence of a bridge between microscopic and macroscopic scales, the missing mesoscopic scale. It is argued that natural selection consequently operates on all scales—whether or not life results will depend on both the initial and the evolving boundary conditions. That life alters the boundary conditions ensures nonlinearity and scale invariance. Evolution by natural selection will have taken place in Earth’s fluid envelope; both air and water display scale invariance and are far from chemical equilibrium, a complex condition driven by the Gibbs free energy arising from the entropy difference between the incoming solar beam and the outgoing infrared radiation to the cold sink of space acting on the initial conditions within evolving boundary conditions. Symmetry breaking’s role in the atmospheric state is discussed, particularly in regard to aerosol fission in the context of airborne bacteria and viruses in both current and prebiotic times. Over 4.4 billion years, the factors operating to support natural selection will have evolved along with the entire system from relative simplicity to the current complexity.

## 1. Introduction

*“Everything that living things do can be understood in terms of the jigglings and wigglings of atoms.”*

Richard Feynman

The origins of life constitute an essentially chemical, and thus molecular, problem. In contrast to many laboratory chemical reaction vessels, however, geophysics ensures an inherent anisotropy and a central, continuing, and evolving role for both initial and boundary conditions in the planet’s fluid envelope, which is perpetually far from equilibrium. While the emergence of biopolymers and living cells in a chemically heterogeneous environment such as the prebiotic Earth presents a formidable problem, both in terms of laboratory experiment and theoretical chemistry, it is worth seeing if any general understanding of the processes, by which the complex products that constitute living matter were produced, can be gained. Here, it is argued that molecular dynamics simulations with the simplest possible representation—that of Maxwellian hard, elastic spheres—offer such an initial clue into the key process of natural selection. If some of its characteristics are evident in such a rudimentary population, they should surely exist in those of real, rotating, vibrating, electronically structured, non-spherical, and chemically reactive molecules, which have attractive as well as repulsive parts of their potential field. The approach is inherently statistical and non-equilibrium, as it has to be, and attempts to synthesize relevant results from the literature into a conceptual argument to yield a fresh perspective. Three characteristics of natural selection in a biotic context that are also present in molecular dynamics simulation of a random population of Maxwellian billiards subject to anisotropic flux are (1) competition for a finite resource—the total energy of the particles, (2) variation in the distribution of that energy—the distribution of velocities, (3) the emergence and propagation of organization, as embodied in the symmetry-breaking hydrodynamic flow with fat-tailed, power law probability distribution that was previously missing in the random sample. During 4.4 billion years after the emergence of the fluid ocean and atmosphere, the emerging, ever-increasing complexity driven by the available Gibbs free energy will change both the chemical composition and the boundary conditions in which natural selection operates, resulting in the added selection criteria evident in current life. These include the role of DNA, the metabolic cycles, the vast population of proteins, and the variability across the biologically influenced fluid envelope.

The technique of simulating molecular dynamics by a numerical process with a computer was invented by Alder and Wainwright [1] and later used to demonstrate the emergence of organized, fluid flow in an initially random (thermalized) population of Maxwellian “billiard ball” molecules subject to an anisotropy arising from the boundary conditions, a directional flux of energetic molecules, Alder and Wainwright [2]. The long, slow power law decay of the velocity autocorrelation function was characteristic, and given a statistical mechanical basis, Dorfman and Cohen [3]. With much larger populations, such calculations have been shown to exhibit scaling behavior, or scale invariance, that is similar to that seen in simulations of the same fluid using the macroscopic continuum Navier–Stokes equations, Kadau et al. [4]; Kadau et al. [5]. In numerical simulations of gases and liquids, it is well-known that the solution of the state by integration of differential equations is determined by interaction with the boundary conditions. Note that this is a dynamical, kinetic approach rather than reliance upon explicit quantum chemical thermodynamics, which requires calculation of the free energies of each molecular species in a population. The scale invariant approach to the Gibbs free energy circumvents that handicap.

The emergence of scale invariance in these simulated molecular populations is important, since it connects microscopic to macroscopic behavior, the molecular world to the biological world. The evidence of “cascades” in fluid systems, Lovejoy and Schertzer [6] supports the idea of interaction on all scales. Cascades in this context have the following characteristics: scale invariance; a scale-by-scale conserved quantity; and locality, the notion that interactions are strongest between, but not limited to, neighboring scales. Scale invariance has a long history in geophysics, from Perrin [7] through Richardson’s [8] observation of power law rather than isotropic Einstein–Smoluchowski diffusion in the atmosphere, to Hurst [9], Richardson [10], and Mandelbrot [11]. Scale invariance has been formulated and used extensively in the theory of statistical multifractals and generalized scale invariance by Schertzer and Lovejoy [12] and by Lovejoy and Schertzer [6] to examine geophysical processes in the fluid volumes of the planet. The formal equivalence of scaling variables in scale invariance to those of statistical mechanics has been known since the 1980s; Lovejoy and Schertzer [6] point out that it goes beyond formal equivalence to a mapping between cascade models and Hamiltonian systems. The flux of energy in the former is equivalent to energy in the latter. We propose that this equivalence provides a potential link between microscopic and macroscopic scales in the atmosphere and biosphere, because the evidence of cascades implies that the same processes operate over the scaling domain, scale by scale. The history of the realization of scale invariance may be traced in references [7,8,9,10,11,12]. The emergence of scale invariance in Maxwellian molecular simulations encourages belief that real molecular populations, with many more degrees of freedom, can span the mesoscopic gap of Laughlin et al. [13] and Laughlin [14]. That subject has been further discussed recently in the atmospheric context [15,16,17] and also applied to microdroplets [18]. Symmetry breaking plays an important role [15,16,17,19,20] and is applicable to atmospheric aerosols [21]. The concept of photostability introduced by Rapf and Vaida [22] has an important role in prebiotic chemistry.

Summarizing references [15,16,17,18,19,20,21,22], it was argued that prebiotic chemistry would have had physically and chemically propitious circumstances for evolution in atmospheric aerosols, which would have sampled a wide range of radiative, temperature, and relative humidity conditions. They would also have interacted with the air–sea interface, with its provision of an anhydrous environment for the condensation reactions needed to eliminate water in the creation of the polymeric amino acids, sugars, and nucleosides. Those reactions are thermodynamically and kinetically highly improbable in liquid water. The aerosols have their own such air–water interface. The interface between the ocean and atmosphere would have acted as an integrator where all the chemicals in the fluid envelope would meet, whether exogenous from meteors, from sea floor vents, or photochemically produced in the air. The breaking of ocean waves would then as now have been a major source of aerosols, with surfactants providing an evolving outer skin containing the aqueous solute and its soluble cargo. Atmospheric aerosols are described by power law size distributions. Such fat-tailed distributions are characteristic of scale invariance and statistical multifractality.

It is noted here that while the original Urey–Miller experiments assumed a prebiotic atmosphere like that of Jupiter to produce the monomers that constitute later biopolymers, recent experimental simulations with prebiotic “air” containing much lower concentrations of methane, ammonia, hydrogen, and hydrogen sulfide show the same behavior [23].

The proposal here offers a way to evaluate the direction in which a non-equilibrium, open macroscopic system will evolve without having to calculate the entropy via conventional methods, which is often difficult to arrive at in real, chemically diverse, macroscopic systems. Recent experimental advances and results seem to offer the possibility of applying the approach to laboratory chemical reactors, Gross et al. [24], as well as to the planet’s fluid envelope, via three or more decades of physical scaling range.

The concept of kinetic stability in chemically reactive systems far from equilibrium has a very long history, Ostwald [25]; Michaelis and Menten [26]; discussed in physical chemistry textbooks, Hinshelwood [27]; Benson [28]; Berry, Rice, and Ross [29] with emphasis on the fluxes of matter and energy across the boundaries in maintenance of the system, giving biochemical examples. This has a similarity to the anisotropic molecular flux causing the emergence of fluid flow in the Alder and Wainwright [2] mechanism, and the complexity of turbulent flow in the atmosphere, Tuck [30]. Randall and Lewis [31] pointed out that the equilibrium state of our nitrogen–oxygen atmosphere above an aqueous ocean was a 0.1 M solution of nitric acid, a non-equilibrium theme further developed by Sillén [32], and by Lovelock [33] who inter alios showed the methane–oxygen ratio in the atmosphere was 30 orders of magnitude removed from equilibrium. Recently, Pascal et al. [34] have developed the dynamic kinetic stability ideas of Pross [35] in the context of the origin of life, emphasizing the importance of exponentially driven self-replication. Autocatalysis has also been an important process, recognized in the historical references at the start of this paragraph and by Pross. It is inherently non-linear and provides a microscopic underpinning for the observed, macroscopic prevalence of generalized scale invariance and statistical multifractality, Tuck [17,30,36]; Lovejoy and Schertzer [6]. It seems appropriate at this juncture to quote the final paragraph of Hinshelwood [37]: “Alternative and competing mechanisms play a great part in chemical kinetics generally and give rise to great complexity. If the spontaneous processes of nature be described in terms of the thermodynamic idea of a descent of energy to lower levels of availability, then we must agree that it does not flow in one placid stream, but rather resembles the water of a mountain lake lashed by the wind to overflow its rocky margin in many tortuous rivulets. The true unity and simplicity are only to be found in the statistical principles according to which the whole intricate system evolves.” A specific and relevant modern exposition is in the vignette by Eisenberg [38] in Chapter 31 of Berry, Rice, and Ross [29].

This paper is constructed by referencing past work, and using it to advance the current topic, the relationship between scale invariance and natural selection as an emergent property originating in the smallest scales—molecules and photons and its relevance to origin of life. References [15,16,17,18,19,20,21,22] offer some of the basic arguments.

## 2. Geophysical Conditions, Prebiotic, and Current

There is significant uncertainty concerning the composition of the early Hadean (pre-4.4 billion years ago) and Archaean atmospheres, together with the oceanic composition and temperature. Continents may have started to emerge in the late Hadean but would have been volcanically produced prior to the later emergence of plate tectonics. The air was possibly mildly reducing in the Archaean, with higher rotation rates arising from the gravitational effects of the proximity of the moon, accompanied by larger tidal effects and breaking waves. Surface temperatures would have been higher, driving storm cloud and lightning activity. The less attenuated solar flux and high lightning activity would have created high concentrations of atoms, free radicals and molecules in excited states, which opened up more possibilities for chemical reactions that would not occur in the present troposphere. The energy flux from the sun, although arguably some 30% less than now, would have exceeded the energy flux from the ocean floor and any crust by one to two orders of magnitude [22]. Enhanced wave activity at the ocean surface would have led to copious production of aerosols. We will return to this subject in the Discussion, Section 8 below.

We note that N_2_ was probably dominant, O_2_ was low but not zero, increasing greatly as a result of the evolution of photosynthesis about 2.4 billion years ago. The reduced gases CH_4_, NH_3_, and hydrogen sulfide become subject to oxidative attack, principally by OH and HO_2_. Strong winds, whitecaps, clouds, and lightning were almost certain, and the length of day was about 18 h over 4 billion years ago. Total pressure is uncertain but may be much lower today than before about 4 billion years ago. Solar UV flux near the surface would have been greater than now, in the absence of an ozone layer, but it is likely that molecules absorbing the UV both acted as shields and evolved from the initiated photochemistry. See references [20,21,22]. Reference [23] is an example where repetition of the Urey–Miller experiment with methane, ammonia, and hydrogen in much lower amounts than in Jupiter still produced the monomers from which biopolymers are made.

**Figure 1:**
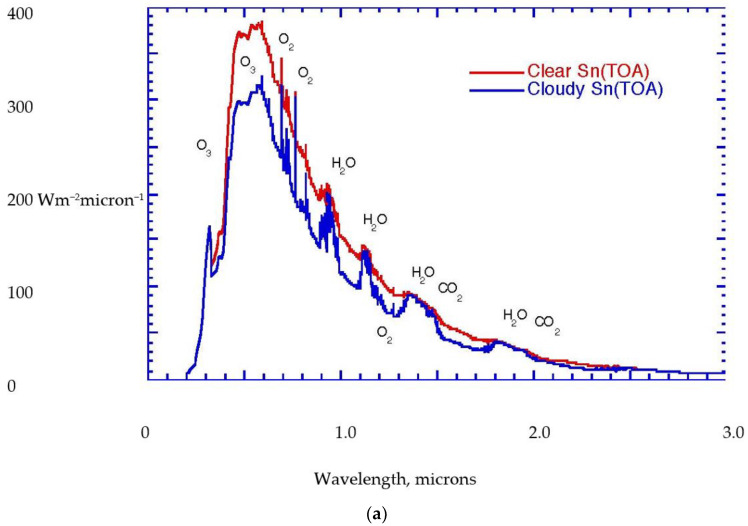

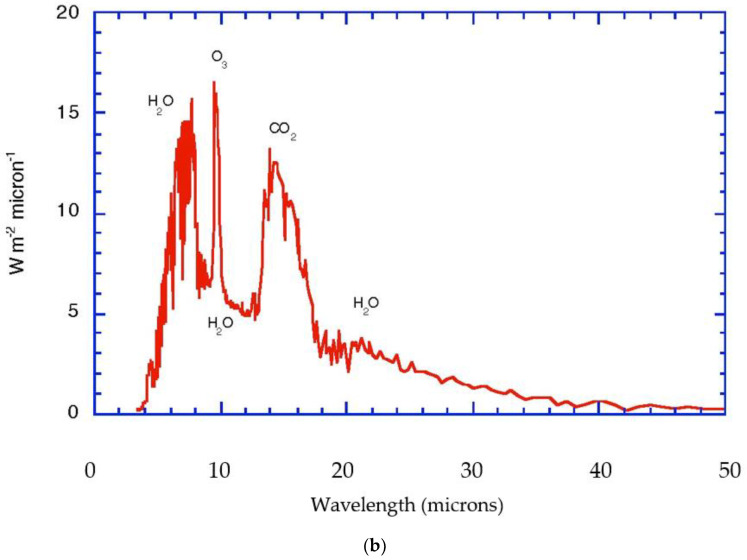
(**a**) Solar flux at the top of the current atmosphere. Red curve for clear skies, blue curve for cloudy skies. The various spectral features are labelled by the molecules responsible. (**b**) Absorbed infrared radiation for the current atmosphere, with the absorption features labelled by the three main absorbers, H_2_O, CO_2_, and O_3_. There are absorptions from N_2_O, CH_4_, and halocarbon molecules in the so-called window region between 7 and 14 microns. After Kiehl and Trenberth [39]. © American Meteorological Society. Used with permission.

**Figure 2:**
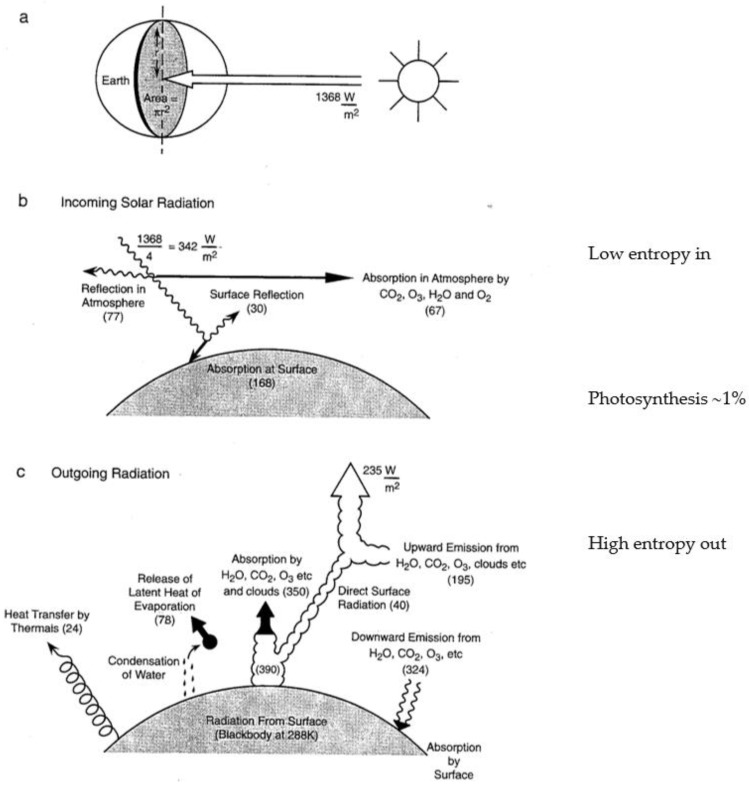
The entropy of a radiative source is Q/T where Q is the energy and T the absolute temperature. The emitting temperature of the sun is about 5800 K, and that of the Earth is about 255 K. Photosynthesis by the entire biosphere is approximately 1% of the solar flux. The units on the figures are Wm^−2^. Note that the currently accepted value for the average influx is 1361 Wm^−2^.

The current atmosphere has been shown to obey statistical multifractality [6,8,12,15,17], which has a thermodynamic form, see Section 3 below. The observed scale invariance can be interpreted in terms of molecular dynamics and related to energetic behavior that parallels that in the biological process of evolution by natural selection [15]. The thermodynamics is formulated as arising from the fluxes of solar and terrestrial radiation, with the incoming beam of solar photons from a source at 5800 K representing an organized state, and the outgoing flux of infrared photons over the whole 4π solid angle from an average temperature of 255 K representing a more disorganized state; possible because the temperature of the sink, space, is 2.7 K. The Gibbs free energy that is made available provides the work that drives the atmospheric circulation [17]. The Gibbs free energy is carried by the most energetic molecules. Figure 1a,b portrays these radiative considerations in quantitative detail for the current state of the planet. Notice that the biosphere uses approximately 1% of the solar flux to drive its photosynthetic activity, Figure 2.

**Figure 3:**
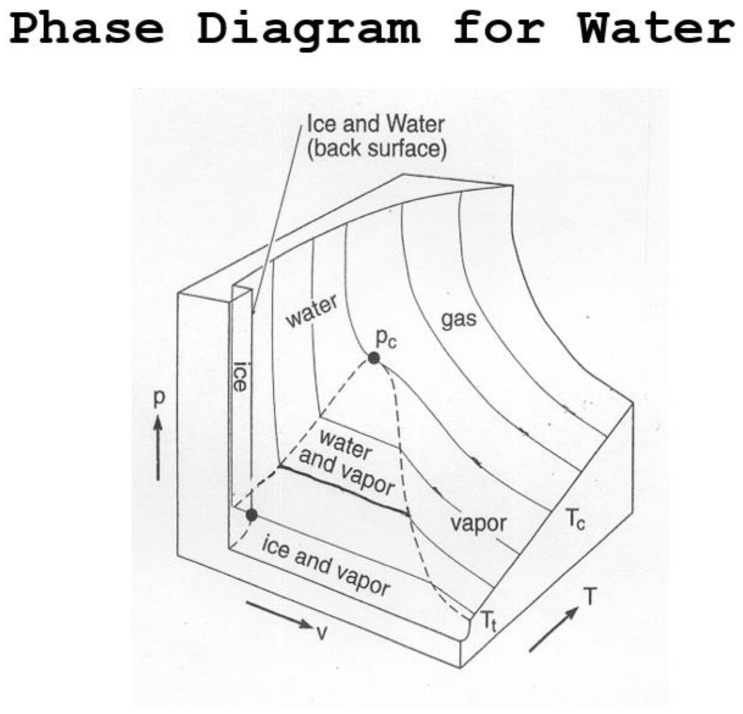
The phase diagram for water shows that the atmosphere can sample all three phases around the triple point, a property unique to water. Ice floats because water has its maximum density at 4C, or 277 K. Water vapor plays a crucial role in establishing atmospheric temperature via the interaction of its phase changes and spectroscopic properties which establish the thermodynamics of the planet. After Vaida and Tuck [40].

The role of water in maintaining the thermodynamic structure of the atmosphere above a liquid ocean has been discussed previously [19]. It is the result of the sampling effects of phase changes of water around its triple point, enabled by its spectroscopic properties interacting with the radiative fluxes described above. The fact that ice floats had and has large implications for the existence of life on Earth, on all scales from the winter freezing of contemporary ponds and rivers to most of the planet during snowball Earth episodes in the Cambrian. Figure 3 illustrates the phase diagram applicable to the present-day planet.

## 3. Scale Invariance

Fractality in biopolymers, specifically in proteins, has similarly long been known, Stapleton et al. [41]. The residue (monomer) sequences in proteins are fractal, a result also seen in nucleic acids, Li and Kaneko [42]; Voss [43]; Peng et al. [44]; Peng et al. [45] with later studies establishing fractality in surface films, Oliveira et al. [46], and membranes, Hoop and Peng [47]; the entropy fluctuation and dissipation in the folding of a hairpin in RNA also obeyed power laws, Crooks [48]; Collin et al. [49]. All these results require the existence of rotational, vibrational, and electronic degrees of freedom, and the attendant potential for chemical reaction, that are not present in the “billiard ball” representation of molecules; the latter however displays some of the properties of a natural selection process, and the emergence of complexity is enhanced by the more numerous degrees of freedom including chemical reaction and the concept of photostability [22]. What is selected in the Maxwellian population is an organized state, emerging with scale invariance from an initially randomized population. In the more realistic populations of real, reactive molecules, natural selection can be seen as an inevitable process. Whether or not it produces life will depend on the initial conditions, and the boundary conditions as they evolve over geological space and time scales. Specifically for example, molecules will not grow indefinitely to produce tars, in, for example, the Fischer–Tropsch synthesis; physical processes and conditions present in the air and sea but not in a typical laboratory reactor will prevent it, such as self-shielding from ultraviolet radiation and the preferential retention of linear rather than branched fatty acids at air–water interfaces, Tuck [21]. Similarly, the promiscuous production of sugars in the Butlerow laboratory synthesis (formose reaction) will lack any sort of useful prebiotic preference (for ribose, to construct nucleotides) unless the initial and boundary conditions offer some organizational direction. The argument is that the emergence of organization results from the initial and boundary conditions in the face of the unceasing dissipation inherent in molecular motion. The Gibbs free energy change is the non-entropic energy, the enthalpy change, carried by the most energetic molecules [15]. Scale invariance is thus a sign that dissipation is effective on the appropriate scaling range; the emergence of organization is not inevitable, but when it occurs it is informative in the sense of a local entropy decrease. Complete randomness would result in Hurst exponent *H* of 0.50; this scaling exponent was originally used to characterize the persistence, or higher-than-random occurrence of positive neighbor-to-neighbor correlation, evidenced by *H* ≈ 0.7, in Nile floods, Hurst [9]; zero would be complete anticorrelation, unity perfect correlation. See also the definition of *H* in Section 3.1 below, in the case where *q* = 1. Note that the terms persistent and antipersistent refer to Gaussian processes, while the atmosphere is shown to be Lévy or multifractal in character [6,15,36].

Organic aerosols have been proposed as a possible vehicle in prebiotic chemistry Dobson et al. [50]; Donaldson et al. [51], and atmospheric aerosols have power law size distributions, Pruppacher and Klett [52], maintained by their coagulation behavior moderated by possible fission processes when they have an organic surfactant coating [51]. The photochemistry of pyruvic acid offers a route in such aerosols to complexity, Griffith et al. [53]. An interesting recent development here is the exploration of microdroplets and their surfaces as accelerators of biophysically relevant reactions, Lee et al. [54]; Fallah-Araghi et al. [55].

There is some evidence of scaling behavior in larger-scale biology, including the metabolic rates of animals as a function of size and the heights of trees, West and Brown [56], patterns of forest fires Turcotte [57], and plankton blooms Seuront et al. [58]. However, with the exception of the plankton, where the turbulent ocean flow is causal, characterization is usually via a single scaling exponent, thereby missing the effects of intermittency and multifractality.

On astronomical scales, scale invariance has been seen in giant molecular clouds, Elmegreen and Falgarone [59]; in saying it is ubiquitous, there is no intent to imply that astronomical clouds had any effect on Earth’s atmosphere at any time. It has been pointed out, Solé and Munteanu [60], that among all known astrochemistries, planetary, and lunar atmospheric chemistries, Earth’s atmosphere is the only one where at least the stratospheric chemistry can be characterized as a scale-free network, a characteristic shared only with cellular metabolic chemistry in the planet’s biosphere. Connections between the atmosphere and biochemistry have been suggested in many places, Miller [61]; Lovelock [28]; Dobson et al. [50]; Tuck [21]; Griffith et al. [53]; Griffith et al. [62], for example.

All these macroscopic non-equilibrium systems present difficulties for formulation of their evolution in terms of entropy, which is a measure rather than a dynamical variable, Grandy [63]. The absence of local thermodynamic equilibrium at even short time and space scales implied by the Alder and Wainwright [2] mechanism means that since reciprocal temperature is defined as the differential of entropy with respect to energy, there is a fundamental difficulty in the context of geophysical fluids, Tuck [28] echoing that pointed out by Gallavotti [64] for microscopic systems. As a result, we suggest that it is worth examining scale invariance in the above phenomena empirically, as a complement to entropy production, rather as the working of steam engines led to equilibrium thermodynamics in the 19th century. Note that there is as yet no rigorous, mathematically complete formalism for the thermodynamic evolution of non-equilibrium systems. There is, however, an invariant formulation of quantum mechanics, Palmer [65], so in principle the range of scales covered could be very many orders of magnitude, from atoms to interstellar molecular clouds.

Other approaches to this formidable problem include entropic ones, Demetrius [66]; Demetrius et al. [67]; Annila and Salthe [68]; England [69], for example, and chemical kinetic networks, for example Kauffman [70]; Kauffman [71]; Jeong et al. [72]; Pross [73]; Caldarelli [74].

We examine similarities and differences between these formulations and ask to what degree they converge to a common approach. An important point of departure is the difficulty of defining temperature and entropy in a non-equilibrium system, pointed out by Gallavotti [64] in a microscopic statistical mechanical context, and for the atmosphere by Tuck [22,28]. The emergence of organization in the form of fluid flow means that dissipation is incomplete in the latter case, and so temperature, in accordance with experience, is of an operational nature rather than being rigorously defined. The price of this procedure is that atmospheric temperature is what is measured by a thermometer, accompanied by a need to understand and formulate the principles upon which the thermometer functions.

Demetrius [66,67] provides a detailed mathematical justification for the equivalence of cell cycle time in a biological entity to temperature in Maxwell–Boltzmann thermodynamics. Given thermostasis, or the absence of measurable temperature gradients in a cell, this seems reasonable. Where the procedure appears to differ from that of scale invariance is in the step to define Demetrius’s evolutionary entropy, which measures the rate at which an organism appropriates energy from its environment and uses it to survive, reproduce and evolve. Directionality then stems from the tendency of evolutionary entropy to increase when resources are diverse and constant but to decrease when the resource is singular and variable. In comparison, in the case of scale invariance, the different macroscopic scales may be regarded as equivalent to the microscopic energy levels, with a derived scaling exponent providing the equivalent to the partition function of Maxwell–Boltzmann statistical mechanics. The fact that it works suggests that powerful physical mechanisms are at work across the range of scales. Directionality is driven by the initial and evolving boundary conditions in what is an open system—in the case of the biosphere, embedded in Earth’s fluid envelope and driven by the entropy difference between the low entropy state of the incoming beam of solar photons and the high entropy state of the outgoing flux of low energy infrared photons over the whole 4π solid angle. This is, interactively with the biosphere, what maintains the profound chemical disequilibrium in the Earth’s atmosphere, the emergence of scale invariant turbulence maintaining the physical disequilibrium. Note that the introduction of time is explicit in the Alder–Wainwright [2] mechanism. Gibbs free energy is the key to directionality of reactions in this approach [15,16,17,18]; it is carried by the most energetic molecules, called chemical potential for individual substances [18].

Autocatalysis has long been known to chemical kineticists, in systems as diverse as the H_2_-O_2_ explosion, branched chain reactions, cool flames, and enzyme kinetics, as referenced above, and applied in a biological context by Kauffman [70,71] to explain the emergence of stable biochemical systems that would be inexplicable on the basis of random reaction probabilities of monomers forming biopolymers. This approach has been extended to make explicit the possibilities entailed by replication through autocatalysis, by for example Pascal et al. [26] and by England [69]. However, as currently formulated it is not clear how sufficient diversity can be generated and maintained, given that a large degree of variation is required for the emergence of organization. Function cannot be anticipated in the progress of evolution, Blomberg [75], who points out that the 20 monomers constituting proteins offer greater variation than the 4 monomers constituting nucleic acids; in any event, current life involves intimate cooperation between the two kinds of biopolymer. Dyson [76] also utilized the greater numbers of amino acids compared to nucleotides to argue priority for proteins and metabolism. The fact that in generalized scale invariance the mean converges but the variance does not, in contrast to Gaussian and Maxwellian statistics, may reflect the requirement for great variation in the population upon which selection operates, see for example Tuck [28], Griffith et al. [53], Lovejoy and Schertzer [6]. The calculation of the two exponents additional to *H* (see Section 3.2 below) in generalized scale invariance, the intermittency *C*_1_, and the degree of departure from monofractality *α* are not specified here; they require a large amount of accurate data that have not so far generally been available for biodata.

This approach seems to be consistent with the remarks about the need for analysis on the mesoscopic scale to link micro and macro scales made by Laughlin et al. [13] and Laughlin [14] and the general arguments about natural selection, evolution, and time dependence made by Smolin [77,78].

### 3.1. Equivalence of Scale Invariant and Statistical Mechanical Variables

It is necessary to relate the thermodynamic form of statistical multifractality to molecular dynamics, and to use the connection to examine natural selection as a process applying to molecular populations. Alder and Wainwright [2] demonstrated that ring currents or vortices emerged from a randomized thermal population of “billiards” subjected to a directional flux of such rigid spheres. Breaking the continuous translational symmetry of the thermal population had produced organized hydrodynamic flow where none had existed previously. It did so on molecular and photon time and space scales.

Dorfman and Cohen [3] analyzed Alder and Wainwright’s result to show that the velocity autocorrelation function, *A*, was not indicative of Maxwell–Boltzmann behavior with an exponential fall-off in the wings of the probability distribution. Rather, it had a fat tail with power law fall-off, indicating long-range correlation and an organized flow condition. They found that *A* was expressed as a function of velocity $\vec{v}$ (*t*) at times *t* in systems of physical dimensionality *d* (*d* = 2, discs; *d* = 3, spheres) by

(1) $$A(t)=d^{-1}\langle \vec{v}(t)\cdot \vec{v}\left(0\right)\rangle \;\propto t^{-d/2}$$

where the angle brackets represent an average. The autocorrelation function, *A*, is a measure of the self-similarity in a series of numbers, times in this instance.

The nonlinear interactions that maintained the emergent, symmetry-breaking fluid flow were conveyed by the most energetic (fastest-moving) molecules. Compared to the pre-existing randomized state, the emergent flow represents an extremely simple form of nonlinearly propagating organization, a primitive memory equivalent. An operational temperature is effectuated by the more nearly average molecules easily exchanging energy in small increments; this dissipative process embodies the necessary entropy production. If a population of Maxwellian billiards is capable of this, a population of real, rotationally, vibrationally, and electronically endowed molecules would be expected to be even more capable of engendering and sustaining complexity, arising from chemical reaction, a necessary but insufficient condition of life.

### 3.2. Equivalence of Scale Invariant and Statistical Mechanical Variables

Following Lovejoy and Schertzer [6], we formulate variable Ψ having data and values expressed as a function of *x*. Ψ can be any observed variable for which the values exist in a regular spatial or temporal series or sequence. In the present case, it is said to be applicable to fluids, membranes, and the monomer residues constituting a biopolymer.

*S_q_* is the *q*^th^ order structure function of Ψ(*x*):

(2) $$S_{q}(r;\Psi )=\langle \left|\right.\Psi (x+r)-\Psi (x){\left.\right|}^{q}\rangle$$

Here, *r* is the lag parameter over the range of *x*. If a graph of log(*S_q_*) against log(*r*) is linear with slope ζ(*q*), it follows that ζ(*q*) is a scaling exponent for Ψ(*x*). In such circumstances, Ψ(*x*) is scale invariant with a power law probability distribution function (PDF).

Let

*H_q_* = *ζ*(*q*)/*q* (3)

with

*H* = *H_q_* + *K*(*q*)/*q* (4)

Here, *H* is sometimes known as the Hurst exponent [9]. Required in the current context are *K*(*q*) and *H.*

*K*(*q*) is found by taking Ψ(*x*) at finite, observed intervals *x* = 1, 2, 3, … and deploying:

(5) $$\begin{array}{l} \varepsilon (1,x)=\left\{\left|\Psi (x+1)-\Psi (x)\right|\right\}/\langle \left|\Psi (x+1)-\Psi (x)\right|\rangle \end{array}$$

with *x* ranging 1, 2, 3, … The individual differences are normalized using division by the mean of unsigned differences in Equation (5).

Next, the following evaluation is performed:

(6) $$\varepsilon (r,x)=\frac{1}{r}\sum_{j=x}^{x+r-1}\varepsilon (1,j)$$

for *x* = 1, 2, 3, …, *x*_max_ − *r*.

From this, a graph of log〈ε(*r,x*)〉^*q*^ vs. log(*r*) is made. It has slope −*K*(*q*). Following that, plotting −*K*(*q*) vs. *q* displays a convex function with *K*(0) = *K*(1) = 0.

Formal equivalence between statistical thermodynamics and scale invariance may now be examined. To do so, energy *E* is defined in the context of a scale ratio *λ* = *L*/*l* where *L* is the outer scale (for example the length of a biopolymer or a great circle around the planet) and *l* is a physically significant scale for the particular case. As a function of λ, the probability of energy *E* is

Pr(*E*_λ_ ≥ *λ^γ^*) ≈ *λ*^−*c*(*γ*)^ (7)

Here, *c*(*γ*) is the co-dimension. It is the difference between the dimension *d* of the “embedding” space and the fractal dimension *D*(*γ*). The latter characterizes the various dimensions of the intermittent singularities. So

*c*(*γ*) = *d* − *D*(*γ*) (8)

Using *K*(*q*) as formulated above

〈(*E_λ_*)*^q^*〉 ≈ λ*^K(q)^* (9)

and

*K*(*q*) = max(*γ*){*qγ* − *c*(*γ*)} (10)

so

*c*(*γ*) = max(*q*){*qγ* − *K*(*q*)} (11)

These equations enable the equivalence between the statistical thermodynamic variables and those of scale invariance to be stated. In Equations (12)–(16) the left-hand side represents the statistical thermodynamics, while the right hand side represents the scale invariant equivalent.

*T* ≡ 1/*q*k_Boltzmann_ (12)

Here, the temperature *T* is the thermodynamic equivalent of the reciprocal of the product of the scaling quantity *q* and Boltzmann’s constant.

*f* ≡ e^−*K*(*q*)^ (13)

The exponent of the negative of the scaling parameter *K*(*q*) is equivalent to the thermodynamic partition function *f*. They describe the distribution of energy over the scales and the molecular energies, respectively.

*E* ≡ *γ* (14)

The thermodynamic energy *E* is equivalent to the exponent to which the scale ratio *λ* is raised, to express the probability *E_λ_*.

*−S*(*E*) ≡ *c*(*γ*) (15)

The thermodynamic entropy, *S*(*E*), is equivalent to the scaling co-dimension, *c*(*γ*) in scaling terms, and *K*(*q*) is defined as above in Equations (4)–(11).

Finally, this permits the Gibbs free energy *G* on the left-hand side of Equation (16) to be defined in scaling terms:

*−G* ≡ *K*(*q*)/*q* (16)

This relation expresses the Gibbs free energy on the left in terms of the scaling ratio on the right. *K*(*q*)/*q* signifies directionality—emergence of organization—in the scaling quantity’s medium, just as *G* does by quantifying the energy available to perform organizing work in conventional statistical thermodynamics. *K*(*q*)/*q* can be calculated in many macroscopic systems. It can be so, moreover, without the need to perform the calculation for each molecular constituent’s chemical potential before summing them, as in quantum chemical thermodynamics. The Gibbs free energy was chosen rather than the Helmholtz version because it is needed in a gas or liquid with ongoing chemical or photochemical evolution: the pressure–volume term is used. In the atmosphere Gibbs free energy is deployed [79], where the entropy is proportional to the product of the specific heat at constant pressure and the logarithm of the potential temperature of dry air, *θ*, as defined by

(17) $$\theta =T{\left(p_{0}/p\right)}^{\kappa }$$

where *κ* = (*c_p_* − *c_v_*)/*c_p_* = 0.288 for dry air. Further terms are required if the thermodynamics of water and its phase changes are to be accounted.

The partition function, *f*, is the quantity describing the distribution of a molecular population over its energy levels. The equivalence in row 2 of Table 1 therefore implies that the scaling quantity e^−*K*(*q*)^ describes the energy distribution over the scales used to analyze Ψ. This relationship could be examined in laboratory systems as well as macroscopic geophysical and biological ones. The experiments such as those of Gross et al. [24], who spatially resolved scales of 10^−6^ meters in a flow reactor of millimetric dimensions, thus permitting the three decades of scaling necessary for reliable scale analysis. Systems potentially open to scaling analysis in these terms include monomer residue sequences in proteins, nucleic acids, membranes, and molecular populations in air and water; all could be assessed for directionality or organization without resorting to the need to compute chemical potentials (Gibbs free energy for individual substances) for each constituent separately.

Table 1 summarizes the equivalences between statistical thermodynamics (column 1) and scaling variables (column 2). Via the calculation of *q* and *K*(*q*) from observational data, the Gibbs free energy can be obtained directly, and hence the amount of energy available to perform work in determining the direction of the evolution.

The Gibbs free energy is related to *K*(*q*) divided by the parameter *q*. The significance of *K*(*q*)/*q* is in its provision of directionality in a scaling environment, in the manner that *G* does in statistical thermodynamics; calculation of it from observation in a macroscopic system means there is a scaling quantity acting like entropy in a quantum thermodynamic system. The pressure–volume work term is used and so selects Gibbs over Helmholtz energy; Gibbs free energy is relevant in fluids with chemical and photochemical reactions.

The variable specifying populations in energy levels in molecular ensembles is the partition function *f*, formally corresponding to e^−*K*(*q*)^ in scaling analysis, as given in Table 1. This says that the varying scales used to analyze macroscopic data perform in the same way as the molecular energy levels of statistical thermodynamics.

The relations in Table 1 are significant in the case here because they enable scaling variables *q* and *K*(*q*) to be obtained for molecular properties, whether in sequences of monomer residues in proteins, nucleic acids, and membranes or in air and water, and to be in place of calculations of entropy production, which can be a problematic measure to calculate in macroscopic, non-equilibrium systems.

### 3.3. Scale-Free Networks

The concept of scale-free networks has received much attention [80,81], giving rise to statistical mechanical approaches to their analysis [82]. A pair of scale-free networks in chemical kinetic reaction mechanisms have been discovered, in stratospheric chemistry [60] and cellular metabolism [83]. Protein–protein interactions in cells have also been observed to exhibit the characteristics of a scale-free network [72,74].

If a chemical kinetic mechanism is analyzed by specifying the reactants as vertices or nodes and the pairwise reaction steps as edges, network characteristics can be analyzed [60]. Via the law of mass action, chemical reaction kinetics is multiplicative and known to produce power law dependences [36,74,84]. Such reaction networks show scale-free behavior when:

*P*(*k*) ≈ *F*(*k*/*L*)*k^−^*^Γ^ (18)

where *P*(*k*) is the probability of a molecule having *k* reaction partners and *F*(*k*/*L*) incorporates a cut-off at some length scale *L*. In this case, “length” means the number of steps between the nodes, all of which are assumed to be of equal length. That is unrealistic chemically, and there will be an inter-event distribution of reaction rate coefficients and the resulting rates. Empirically, it is often found that 2 < Γ < 3 [60]. The law of mass action often does not apply, for example in the gravitationally stratified stratosphere [36,84] and the crowded cellular interior [83]. The chemical kinetic rates and their power law dependences must be determined in each case.

In the stratosphere [84] and the crowded cellular medium [83], the law of mass action may not apply. Quantum molecular dynamics simulation would be the fundamental approach but may face insuperable technical difficulties and computational requirements, although this situation may be changing [16]. The approach supplied here is open to empirical, observational analysis, as shown for chemical systems as diverse as the two examples above. Its application to one-pot laboratory experiments aimed at prebiotic synthesis looks possible. Synchrotron radiation has been used to resolve 10 micrometer scale reactant concentrations in a centimeter scale flow reactor—for example, Gross et al. [24]. The dynamic stability shown by scale-free networks is owed to a few chemically promiscuous species, which offer many paths through the network, thereby offering a degree of buffering against perturbation—stable but not invulnerable.

## 4. Natural Selection

The central argument of this paper is that natural selection is a process which is inherently operative in molecular populations possessing favorable initial conditions and boundary conditions that are open and that allow fluxes of energy—as radiation or mass—inward and outward [15]. Symmetry breaking is an important aspect [16,17] and applies in the current context to bacterially and virally sized particles [18,19,20]. Gibbs free energy is a key thermodynamic quantity, enabling work to be done to maintain states that are removed from equilibrium [17]. Carried by the most energetic molecules, it breaks the continuous translational symmetry of equilibrated populations to produce scale invariance [17]. Fission and fusion of aerosol particles with organic coatings is a possible manifestation of the operation of natural selection in the current context [50,51,53]. The three features of molecular behavior at the simplest level—variation, competition, and propagating, organized response to a symmetry breaking flux—are tenets held to be characteristics of natural selection on all scales, including a biological context. The propagating organized response is viewed as an extremely primitive form of memory, the distant forerunner of the DNA-propagated code of biotic reproduction. The process, natural selection, produces the product, life. The environment in which the selection operates will share in the transition from relative prebiotic simplicity to current biological complexity, on geological timescales. The principle and criteria of natural selection evolve over geological time scales.

### 4.1. Emergence

Emergence stems from the mesoscale, intermediate between the microscopic (photons, atoms, and molecules) and the macroscopic (tens of nanometers to astronomical). In the case of the atmosphere, the need to account for reproducible behavior [13,14] at the “middle way”, the mesoscale, is attributed to the action of molecular velocities in defining an operational temperature [15,36] so that in the emergence of ring currents (fluid mechanical flow) the entropic price is paid by the energetically near-average molecules, while the most energetic provide the work to maintain the organized flow. Many biomolecular polymers span meso to macro scales.

### 4.2. Symmetry Breaking

A thermalized population of molecules has a Maxwell–Boltzmann distribution of velocities and possesses continuous translational symmetry [17]. In the atmosphere, that symmetry is broken by the persistence of molecular velocity after collision [16] on the smallest scales and propagates upscale within the lower symmetry of scale invariance. The ever-present anisotropies in the atmosphere—solar flux, planetary rotation, surface topography, and gravity—ensure that isotropy is never observed [17,36]. In the context of biopolymers, once a small oligomer has formed, the environment for its continued growth will be asymmetric, particularly at the air–water interface [19,20,53].

## 5. Complexity

There is no rigorous definition of complexity, but it is immediately evident when examining the behavior of a megadalton protein or nucleic acid. Moving upscale, the complexity persists on all scales to that of the entire biosphere [21,50,51,53]. Complexity is generated if the occurrence of an event—say the addition of a monomer to an oligomer—increases the probability of its repetition in some similar but not necessarily identical form. In the case of a protein, there could be 19 similar but not identical extenders of the carbon chain; in the case of a nucleic acid, three nucleotides are the alternative. Given the number of monomers in typical proteins and nucleic acids, the possibilities are very large, as observed. The variety of monomers provides the symmetry breaking necessary to avoid the exact repetition that would lead to Schrödinger’s problem of the lack of information storage inherent in a perfect crystal [85]. The environment in which biomolecules operate, from within the cell to the planet’s fluid envelope, is characterized by the inherently nonlinear character of the flow, further sustaining the complexity. The fat tails of the probability distribution functions associated with scaling behavior signal nonlinearity and long-range correlation. Those are the characteristics of organized complexity [15,53,56]. Gibbs free energy enables the work necessary to maintain, naturally select, and evolve the systems, on all scales. A particularly relevant scale in the present context is that of microparticles, including atmospheric aerosols [18,19,20].

A further aspect of complexity is that the multiple nonlinearities inherent in the system we are considering frustrate any approach to ergodicity [86] and hence make maximum possible entropy production difficult to attain.

## 6. Peptides, Nucleotides, Proteins, and Nucleic Acids

Evolution by natural selection is made more probable when there is a large degree of variability in the subject population [75]. Directionality is determined by the availability of Gibbs free energy to enable the requisite work. The direction cannot anticipate function [21,75] and will be determined by the detailed molecular dynamics, as will therefore any early metabolic pathways. Current living cells are approximately 70% liquid water and are therefore a scale invariant medium, which enables the Gibbs free energy to be calculated by obtaining it without recourse to treating all the molecules present to individual quantum statistical equilibrium thermodynamical analysis [15,18]. The number of different amino acids available is in the many hundreds [87], of which 20 are involved in being selected for peptide and protein synthesis. The same principles apply to the selection of purines and pyrimidines for the construction of oligomers and nucleic acids, with only three being common to both RNA and DNA plus two others each of which is found only in one or the other of the two acids. Co-evolution of peptides and nucleotides to early proteins and nucleic acids seems highly probable given the degree of variation that must have been present in the prebiotic chemical milieu, together with the associated variation in functions such as photon absorption, energy transport, and storage. Photon transport in an aerosol would be enhanced via the lengthened photon mean free paths and is a very complex process in current phototrophs. Such activity would have been promoted and facilitated by water–air interfaces in aerosol particles, themselves present in enormous numbers subject to varying fields of irradiation, temperature, pressure, and relative humidity [17,19,20,21,50,53,62]. These aerosol populations would themselves have been subject to evolution by natural selection. Peptide bond formation has been directly observed at the air–water interface [88].

The number of vibrations in a nonlinear molecule containing *N* atoms is 3*N* − 6, where there are also three translational and three rotational degrees of freedom. This provides an enormous number of opportunities for chemical interaction, and for directionality to emerge there must be both some form of “memory”—propagating organized complexity—and exposure of sites for continuing the chemical reaction. These are provided, respectively, by DNA and many proteins that are cooperatively active in the operation of current-day cells. This is, of course, a rapidly changing area of research, involving extremely complex processes and such controversial topics as epigenetics and its mechanisms. However, a very simple, coarse picture can be offered: the DNA double helix has its relatively inert phosphate/sugar backbone exposed outside, with the information-carrying bases protected inside. Many but not all proteins fold so that reactive and chromophore groups, usually polar, are in contact with the aqueous medium, exposing groups able to perform such tasks as enzymatic catalysis and binding to nucleic acids and other small molecules involved in cellular functions. That does not include those proteins that perform tasks that involve them being folded into configurations where active centers and groups are internal rather than external, such as many enzymes. Over geological time, many foldings, unfoldings, captures, and releases of co-factors could have occurred.

## 7. Bacteria and Viruses

It has been shown that the Miller–Urey experiment when repeated so as to generate aerosols produces enhanced yields, both quantitatively and in terms of the variety of amino acids and nucleosides [23]. That enhances the earlier suggestion that atmospheric aerosols could have functioned as prebiotic reactors [18,21,50,51,62]. Recently, it has been shown that lightning produces copious quantities of excited and charged species, not just reactive nitrogen [89] but water-based HO_x_ as well [90]. That would increase the possibilities for chemical diversity via reaction with the major constituents. Recently, it has been reported that lightning produces small aerosols [91], and that excited states arising from the three-body photodissociation of water could have produced molecular oxygen in the prebiotic atmosphere [92]. Both processes would have further potentiated the emergence of prebiotic precursors of life.

It was noted that a surfactant coating on an aerosol particle could potentiate fission, a possibility not available in a homogeneous particle [51]. It was further noted that the fission would produce asymmetric daughters, with the larger one being bacterially sized and the smaller one virally sized; it is an example of the role of symmetry breaking. Depending upon the molecular scale processes, chemical differentiation could also occur between the daughters, with the surfactants going to the larger particle, with the smaller particle being coated with any oligopeptides available. This possibility could be tested by experiment, or molecular dynamics calculation. Viruses may be an intermediate state between the undoubtedly living single-celled bacteria and the unambiguously dead state of a population of molecules that would result when a virus is disassembled into its constituent molecules. A single-celled bacterium is an organized, open non-equilibrium system capable of autonomous reproduction. An isolated virus is an organized closed equilibrium system [70,71] incapable of autonomous reproduction. Context may be enhanced by consideration of the arguments in [51].

It has been argued and shown that microparticles, including aerosols, facilitate and accelerate chemical reactions, especially those involving organic molecules [18,19,20,21,40,50,51,53,54,55,62,93,94,95,96,97,98,99,100,101,102]. The effects can occur both at the interface and within the particle, arising from the anhydrous nature of surfactant films, which can carry electric charge, and from increased concentrations of reactants within the interior.

The necessity of containment of the reactants in the prebiotic fluid envelope has long been recognized as required to limit the diluting effects of diffusion and hydrodynamic flow [21,50,51,93,99,100,101]. The fusion and fission of microdroplets would have enabled horizontal transfer of contents in the prebiotic fluid envelope and early biosphere, offering a basic physicochemical mechanism for the horizontal gene transfer posited by Woese [102] and the symbiosis following engulfment to produce organelles as proposed by Margulis [103].

Current atmospheric aerosols contain biological material [104,105,106,107] as well as organic species [50,51,108,109,110]. Reference [108] was inspired by the observations in reference [109] and [50] was in turn inspired by [108]. The basic physical chemistry and meteorology strongly indicate that aerosols would have behaved in the same way in prebiotic times as they do now, especially at the ocean surface [19]. In the current context, observations suggest that the SARS-CoV-2 virus is airborne [106] and that processes are active in open air that modify their transmissibility [107]. The extent of the water interior concentration and of the “sheath” around airborne viruses appears to be of central importance [110]. The relative humidity in mammal lungs is normally 100%, but in most of the atmosphere that is not the case, fluctuating between 2% and 100%. The chemical interaction between airborne microbes and the reactive atoms, free radicals, and molecules that exist photochemically in air is likely to result in changes that will amount to evolution by natural selection. The Outside Air Factor [107] is probably linked to such phenomena and the relative humidity fluctuations. The process was probably active in prebiotic and early biotic eras [21].

## 8. Discussion

Molecular dynamics calculations of equilibrated (thermal) populations of Maxwellian “billiard balls” subjected to a symmetry-breaking flux [2] have shown three characteristics that are the basic principles on which natural selection is based: (1) competition for a finite resource—the collisional partitioning of the translational energy of the molecules, (2) variation—the fat-tailed probability distributions of those energies, and (3) memory—the emergence and propagation of organization, embodied in the previously absent hydrodynamic flow) [17]. These three properties arise in this simplest of simulations of molecular behavior, remarkably enough. For real molecules, the rotational, vibrational, and electronic degrees of freedom will amplify the emergent possibilities for organization, chemical reaction, and the associated complexity. Further consequences follow from the fact that the organization results from nonlinearities in the behavior of the fastest moving—most energetic—molecules, while entropic dissipation by efficient energy exchange among the numerous molecules near the average results in an operational temperature. A significant result of such calculations is that scale invariance emerges, enabled by the Gibbs free energy carried by the most energetic molecules. Negative entropy production by the energetic molecules yields organization, while the more probable molecules produce positive entropy which maintain temperature. Seen this way, natural selection is a process native to molecular populations, resulting in evolution if the initial and boundary conditions permit. Natural selection has been described long ago as a physical process by Lotka [111], but acting on macroscopic scales rather than being rooted in the molecular ones appealed to here. Harte [112] has used maximum entropy analysis in ecological systems. The “evolutionary entropy” approach has been pioneered by Demetrius and collaborators [66,67] and has been compared to our scaling approach in Section 2 above. There are many “entropies” for non-equilibrium systems defined in the literature.

Scale invariance appears on all scales on Earth, from the mean free path at the surface to a great circle [6,12,15,17,18]. It is seen internally in monomer sequences in proteins [39,113,114]. This result is also seen in monomer sequences in nucleic acids [40,41,42,43] and for example [115]. Fractality and scale invariance are evident in surface films [44] and membranes [45]. Entropic fluctuation–dissipation theorems have been observed for the folding and unfolding of a hairpin motif in an individual RNA molecule [46,47]. Scale invariance has also been established in biospheric and ecological systems; see for example Harte [112]. Atmospheric aerosols show power law size distributions and may play a role as prebiotic chemical reactors [46,47]. Fat-tailed PDFs and fractal scale invariance have been analyzed in giant interstellar molecular clouds, location of the processes originating stars and solar systems [59]. Isotropic turbulence is absent on all scales in the atmosphere [116]. The law of mass action cannot be applied with rigor to atmospheric chemical reactions, since the reactants do not have free random access to the entire reaction volume [16,83,84,86]. Scale invariance may be useful as a complement or alternative to entropy production as a measure in analyzing the evolution of non-equilibrium systems. It could apply in understanding the outcomes of one-pot laboratory experiments to form biopolymers in simulated prebiotic conditions [117], or to investigations of the biosphere and atmosphere. As a final observation on this topic, Lovejoy and Schertzer [6] have shown scale invariance in a space-time analysis of weather and climate yielding perspective to the separate spatial and temporal results. Weather, macroweather, and climate act as agents of natural selection. A maximum entropy production approach to the statistical thermodynamics of the atmosphere has been given by Kleidon [118], which may be given a scale invariant context by the thermodynamic formulation of statistical multifractals via *K*(*q*).

Due to the differing perspectives offered upon entropy by the present scaling theory, by evolutionary entropy theory [66,67], and by dynamic kinetic stability theory [73], we consider how entropy can be viewed in the context of scale invariance. The co-dimension of energy in scaling theory maps to the negative of entropy in Hamiltonian systems; see Table 1. Note that entropy is used rather than information. Entropy is directly calculated from dynamical variables, whereas information is derived as a measure from a probability distribution, a procedure that is indirect and entails a degree of subjectivity [119,120], as does the selection of priors. The need to choose coarse graining entails further subjectivity. Information is a widely used concept, but it is not clear how to use it in the approach adopted here.

The mapping shown in Table 1 between the scaling exponent e^−*K*(*q*)^ and the partition function *f* says that the array of scaling intervals used in analysis of macroscopic observations operate like the microscopic energy levels of Boltzmann’s statistical mechanics, indicating that the same processes are at work scale-by-scale over the whole scaling range. Organization and direction are native at the molecular level via the Alder–Wainwright [2] mechanism, interactively evolving as constrained by the initial and boundary conditions, with the boundary conditions altered by the system itself (the emergence of photosynthesis and an oxygenated atmosphere about 2400 million years ago is an example). The molecular behavior therefore underpins both evolutionary entropy and dynamical kinetic stability, which are higher-level formalisms.

The view offered here also resolves the controversy described by Mitchell [121] generated by Kauffman’s [70,71] argument that organization can emerge via random Boolean networks of chemical reactions, without the operation of Darwinian natural selection. The perspective here is that Darwinian natural selection is a mechanism operative in the simplest representations of non-equilibrium molecular populations subject to anisotropic boundary conditions. It does not of course follow that the emergence of life is inevitable, the process does not always result in the living product—the initial and boundary conditions must be favorable. The emergence of turbulent flow from such populations [17,27] also underpins the biological scaling arguments of West and Brown [56] and Krakauer et al. [122] given that such flow is ubiquitous in the planet’s fluid envelope in which the biosphere is embedded and in the internal circulatory systems of cells, plants, and animals. Finally, the possibility of the non-convergence of variance and of *K*(*q*)/*q* (Table 1), the mapping of Gibbs free energy, means that a complete, directional evolutionary theory with no exceptions may be an elusive goal. The air–sea interface would have acted as an integrator for organic molecules, whether they were from ocean floor vents, from space, or produced photochemically in the air. The production of aerosols at the sea surface would have ensured their inclusion in the vast populations continually produced by breaking waves [19]. Lightning produces aerosols [92] as well as reactive nitrogen [90] and reactive hydrogen [91], with photochemistry producing excited oxygen species from water [123]. Photostability would have been an important consideration [22].

Organization emerges in a molecular population by the interplay of the most energetic molecules with the boundary conditions, in competition with the entropy-producing effect of the more probable, near-average molecules maintaining an operational temperature. While the result is for simple Maxwellian “billiard balls” it is argued here that similar considerations apply to the rotational, vibrational, and electronic energy modes, and the van der Waals forces in real molecules in supplying activation energies needed to permit reaction. It is necessary to be specific for both the mechanisms for producing organization and those producing dissipation in non-equilibrium molecular populations, a conclusion reached for macroscopic systems in Volk and Pauluis [124] and also touched upon in Vaida and Tuck [40].

Stratospherically, analysis of observations of the mole fractions of chemical species has shown the value of *H*_1_ to be diagnostic for whether it is a passive scalar (a tracer), has a source, or has a sink, if *H*_1_ = 5/9, *H*_1_ ≥ 5/9 or *H*_1_ ≤ 5/9, respectively [15,17,36,84,125]. It would be of interest to apply this diagnostic to laboratory results. The value of 5/9 arises from the vertical scaling of temperature in the atmosphere, see [6,17,36]. Its value for other media and situations would need to be determined independently, as would the values for particular observations of a given chemical system. The ability to determine whether a particular reactant is being produced (source), destroyed (sink), or is left unchanged could be useful in many reactive situations, whether in laboratories or elsewhere.

Earth’s stratospheric chemistry and cellular metabolism are both scale-free networks [60], reinforcing the argument [15] that the large, continuing chemical disequilibrium in the planet’s atmosphere is caused by its role as an integral part of the biosphere. The presence of viable bacteria and viruses in the atmosphere has long been known, since Tyndall’s discoveries in the 19th century, but modern observations have established their presence in upper tropospheric jet streams, enabling transport across the Pacific Ocean from Asia to North America, Burrows et al. [107]; Smith et al. [108]. A corollary of this result is that airborne microbes will be subject to attack by the primary atmospheric oxidizer, the OH free radical, OH, and its partner, HO_2_ will have been agents of natural selection from prebiotic times, since the solar spectrum then would have had wavelengths capable of photodissociating water vapor penetrating well down into the lower atmosphere [21,22]. Fluctuations of relative humidity would also have been an agent of natural selection [110]. The presence in the atmosphere of such reactive chemical species as OH and HO_2_, which offer many pathways in the reaction network to achieve a given result, cause the stability observed in scale-free networks. A similar role in the citric acid cycle of cell metabolic chemistry is played by pyruvic acid, embodying the concept of driver reactions of relatively small molecules [126], that provide the necessary chemical promiscuity. The observed but rare intermittent occurrences of rapid instabilities under boundary condition changes happen as nonlinear couplings rapidly amplify via the product of reactive concentrations. They are the cause of the fat tails in the PDFs. A recent example is the Antarctic ozone hole [84,127], and there are others in the planet’s past, for example transitions between interglacials and ice ages which are close-coupled to sudden changes in atmospheric CO_2_ mole fraction [127]. Mass extinctions are well-known features in the fossil record, see for example Brasier [128].

Further work on the application of scale invariance and multifractality can be found in Fallico et al. [129] and Zimmerman and Tartakovsky [130], which discuss flow in aquifers and solute behavior in networks. The current state of climate modelling in the light of statistical multifractality is given in Lovejoy [131]. A technical account of information theory as an alternative to entropy can be found in Curilef and Plastino [132]. The work discussed here treats the atmosphere using specific heat at constant pressure rather than specific heat at constant volume, an approach that is standard in meteorology [79]. For a possible alternative approach, see Kamer et al. [133].

A further point may be made about symmetry breaking. For example, it is often assumed that all monomer reactions to produce biopolymers proceed at the same rate, but it has been observed that reactions of pairs of the 20 amino acids to form dipeptides concerned are anything but random [134]. The side chains may be capable of inducing feedbacks, resulting in preferred terminal sites for reaction with preferred monomers, thus breaking the continuous translational symmetry of a random mixture.

## 9. Conclusions

Natural selection appears to be a process inherent in even the simplest molecular populations subject to an anisotropic or dynamic environment, if starting from suitable initial conditions. The products of the process appear to be scale invariant: whether or not life results would have depended upon the evolving boundary conditions, with the emergence of scale-free chemical reaction networks being evident only in Earth’s atmosphere and cellular metabolism, so far at least. If this view has merit, it is implied that life is a product of natural selection rather than vice versa—the process produces the products, and does so on all scales up from the smallest, that of photons and molecules. Fat-tailed probability distributions and great variation are produced by cascades of multiplicative processes operating on all scales, up from the smallest. These statistical multifractal properties mean that while selection operates on all scales, the prediction of what characteristics will be selected for at each level becomes an indeterminate, particularly as the variance does not converge. We note that turbulent flow is present both inside and outside organisms and is scale-invariant [17,18]. Symmetry breaking plays an important role [16]. The entropic dissipation associated with the production of the organizing Gibbs free energy defines an operational, non-equilibrium temperature [15,17]. The non-convergence of variance is consistent with large changes and the generation of complexity.

Natural selection operates on all scales through cascades of the multiplicative processes characteristic of statistical multifractality. Scale invariance has the potential to be a means of studying the evolution of reaction networks in non-equilibrium molecular populations, in laboratory, biospheric, and geophysical systems. The operation arises from symmetry breaking and the work done by Gibbs free energy, ultimately derived from the solar irradiation and the entropy production dumped by infrared radiation to the cold sink of space.

## Figures and Tables

**Table 1:** Equivalence between statistical thermodynamic and scaling variables.

Variable	Statistical Thermodynamics	Scaling
Temperature	*T*	1/*qk*_Boltzmann_
Partition function	*f*	e^−*K*(*q*)^
Energy	*E*	*γ*
Entropy	−*S*(*E*)	*c*(*γ*)
Gibbs free energy	−*G*	*K*(*q*)/*q*
